# Activity-Based Algorithm for Translating Observed Outpatient Activity into Specialist Working-Hour Requirements Within Community Health Center Catchment Areas

**DOI:** 10.3390/healthcare14152435

**Published:** 2026-08-06

**Authors:** Edoardo Trebbi, Tommaso Barlattani, Antony Bologna, Livia Tognaccini, Massimo Maurici, Antonio Vinci, Giuseppe Di Martino, Cristinel Stan, Camillo Odio, Tommaso Staniscia, Francesca Pacitti, Ferdinando Romano

**Affiliations:** 1 Department of Public Health and Infectious Diseases, “La Sapienza” University of Rome, 00100 Rome, Italy; edoardo.trebbi@uniroma1.it (E.T.); livia.tognaccini@uniroma1.it (L.T.); ferdinando.romano@uniroma1.it (F.R.); 2 Department of Biotechnological and Applied Clinical Sciences (DISCAB), University of L’Aquila, Via Vetoio, 67100 L’Aquila, Italy; antony.bologna@graduate.univaq.it (A.B.); francesca.pacitti@univaq.it (F.P.); 3 Department of Biomedicine and Prevention, Tor Vergata University of Rome, 00133 Rome, Italy; maurici@med.uniroma2.it (M.M.); antonio.vinci.at@hotmail.it (A.V.); 4 Department of Medicine and Ageing Sciences, “G. d’Annunzio” University of Chieti-Pescara, 66100 Chieti, Italy; giuseppe.dimartino@unich.it (G.D.M.); tommaso.staniscia@unich.it (T.S.); 5 Local Health Authority ASL 1 Avezzano–Sulmona–L’Aquila, 67100 L’Aquila, Italy; cstan@asl1abruzzo.it; 6 Department of Health, Information Flows, and Digital Health Service, Abruzzo Region, 65121 Pescara, Italy; camillo.odio@regione.abruzzo.it

**Keywords:** activity-based workload model, community health centers, health workforce planning, hub-and-spoke model, Italy, Ministerial Decree 77/2022, outpatient cardiology, specialist working hours, territorial health planning

## Abstract

**Highlights:**

**What are the main findings?**

- Converts observed outpatient cardiology volumes into specialist working-hour requirements for each hub-and-spoke node.
- Links municipal-level activity data, standardized procedure times, and catchment assignments within an Italian community health center network.

**What are the implications of the main findings?**

- Provides a transparent activity-based workforce-planning layer for implementing Ministerial Decree 77/2022 in low-density and mountainous settings.

**Abstract:**

**Background/Objectives:** In Italy, Ministerial Decree 77/2022 provides the regulatory framework for reorganizing community-based care around community health centers and hub-and-spoke territorial networks. However, translating territorial allocation into specialist working-hour requirements remains a key operational challenge, particularly in low-density and mountainous areas. This study developed an activity-based workload model to translate observed outpatient cardiology activity into estimated specialist working-hour requirements within the hub-and-spoke community health center network of local health authority (ASL 1) Avezzano-Sulmona-L’Aquila, Abruzzo, Italy. **Methods:** A second-stage workforce planning analysis was conducted using the baseline 30 min territorial configuration from a previously defined allocation framework. Municipal-level outpatient cardiology administrative data for 2019 were linked to standardized procedure scheduling durations and aggregated across the catchments of three hub centers and eight spoke centers according to predefined municipality-to-node assignments. Procedure volumes were converted into annual and weekly workload hours and then into nominal 36 h weekly specialist-schedule equivalents. The resulting estimates represent a pre-pandemic, utilization-based workload scenario derived from observed activity; they should not be interpreted as direct measures of underlying population demand, epidemiological need, or unmet need, nor as validated staffing requirements. **Results:** The reference network covered 286,832 residents and included 44,039 outpatient cardiology procedures, corresponding to 12,719.8 annual workload hours. These volumes generated 244.6 weekly workload hours, equivalent to 6.79 nominal 36 h weekly specialist-schedule equivalents. Workload was unevenly distributed across the network: the L’Aquila and Sulmona hubs accounted for the largest annual workloads, with 3014.9 and 2894.3 h, respectively, whereas Trasacco was the spoke center with the highest observed workload, with 1775.5 h. Population-standardized indicators showed substantial variation in workload intensity across the catchments, indicating that catchment population size alone did not adequately capture the observed workload differences. **Conclusions:** Activity-based workload estimation adds an operational workforce layer to hub-and-spoke territorial planning. By translating observed outpatient service-use volumes into specialist working-hour requirements, the model provides a transparent and potentially transferable framework to support the operational planning of community health centers in geographically complex settings. The approach is adaptable to other specialties, territories, and service configurations where comparable activity data, standardized scheduling durations, and territorial assignment inputs are available.

## 1. Introduction

Health systems are increasingly required to respond to the aging population, the growing burden of chronic diseases, workforce shortages, and persistent territorial inequalities in healthcare provision [1,2,3]. These pressures are especially relevant for primary and community-based care, where continuity, coordination, and timely access are central to managing chronic and complex conditions [4]. In rural, remote, and mountainous areas, access is shaped not only by the physical presence of facilities but also by travel time, transport conditions, workforce availability, and the capacity of local services to meet population needs [5,6].

In Italy, these challenges intersect with long-standing regional disparities in the organization, availability, and accessibility of healthcare services [7,8,9]. Ministerial Decree (DM) 77/2022, within the framework of the National Recovery and Resilience Plan, introduced a new model for territorial healthcare based on community health centers, community hospitals, and territorial operations centers [4,10]. Community health centers are organized using a hub-and-spoke model and are expected to serve as access points for primary care, nursing care, prevention, chronic disease management, social–health integration, and selected specialist outpatient services [10,11]. However, implementation remains uneven across regions, partly because the Italian National Health Service combines a national regulatory framework with substantial regional autonomy [7,12].

A key unresolved issue concerns the missing link between territorial service allocation and the professional time required to make these networks operational. Although DM 77/2022 defines structural and organizational standards for territorial care, recent Italian studies have mainly focused on locating community healthcare facilities and assessing their accessibility impact, while outpatient specialist working-time requirements remain less clearly operationalized [13,14,15]. This issue is particularly relevant because outpatient specialist care, general practice and pediatrics, are pillars of community healthcare in Italy and play important roles in managing patients with chronic conditions outside hospital settings. However, detailed data on outpatient specialist workforce input, delivered hours, booked visits, and waiting-list dynamics remain limited, making it difficult to estimate actual service capacity and outpatient specialist workforce requirements in community settings [15]. Recent Italian work has applied allocation algorithms to assess access to cardiology services at the national level; however, less attention has been paid to translating observed outpatient cardiology volumes into node-specific specialist working-hour requirements [16].

Health workforce planning should move beyond simple headcounts or population-to-provider ratios and incorporate population characteristics, service utilization, workload, geography, skill mix, and models of care [2,3,17,18,19]. In rural and low-density areas, centrally defined standards may be insufficient if they are not adapted to local travel times, settlement dispersion, workload intensity, and local patterns of service use [3,5,6]. This is particularly important for outpatient specialist services, where professional shortages and uneven distribution can lead to delayed access, longer waiting times, greater reliance on private expenditure, and unmet care needs [15,20].

The Province of L’Aquila, within the local health authority (ASL 1) Avezzano–Sulmona–L’Aquila, provides a relevant setting for testing a workforce-oriented planning approach. It is a large inland and mountainous territory, characterized by dispersed settlements, low population density, and substantial travel-time barriers to community-based healthcare access. For example, Campotosto and Alfedena—the northernmost and southernmost municipalities of the province, respectively—are approximately 169 km apart, with an estimated car travel time of nearly three hours according to the 2021 Italian National Institute of Statistics (ISTAT) origin–destination road travel-time matrix. This illustrates the spatial scale and internal accessibility constraints of the area. Abruzzo is also characterized by a complex spatial organization of inner areas, where planning essential services requires attention to functional relationships, proximity, and everyday accessibility [21]. In such inland and mountainous contexts, dispersed settlements and travel-time constraints may affect access to community-based services, making alignment between service location and workforce allocation particularly important [6].

A previous territorial planning study in the Province of L’Aquila developed a capacity-constrained hub-and-spoke allocation algorithm to assign municipalities to three hub nodes and eight spoke nodes, using official ISTAT road travel-time data and population-based capacity constraints. The study showed that full territorial allocation was feasible under a 30 min benchmark, but proximity-based allocation could overload the L’Aquila hub, requiring a trade-off between travel-time accessibility and workload distribution [22].

These findings establish the territorial basis for a second, still unresolved planning question: once municipalities have been assigned to hub-and-spoke nodes, what specialist working time is associated with the observed outpatient activity attributed to each center? Existing studies on community health centers have primarily addressed facility location, geographic accessibility, and territorial allocation, whereas the translation of observed outpatient service-use volumes into node-specific, activity-derived working-hour estimates remains limited [13,14,15,16,22]. This gap is operationally relevant because territorial allocation alone does not quantify the professional time associated with the observed workload assigned to each node [2,3,15,17,18,19].

Cardiology is an appropriate initial clinical application for this second step. Cardiovascular diseases remain a leading cause of morbidity, mortality, hospital admissions, and healthcare expenditure in Europe, and their management requires effective integration of prevention, primary care, specialist outpatient assessment, diagnostics, follow-up, and post-acute continuity of care [23,24,25]. In community settings, outpatient cardiology capacity is important for chronic disease monitoring, risk stratification, timely referral, and continuity after acute events [20]. At the same time, outpatient cardiovascular care is sensitive to territorial variation in visit rates, waiting times, and local service availability, as shown by regional analyses of cardiology visit rates and waiting times in Tuscany and by broader evidence on outpatient specialist workforce capacity in Italy [15,20].

Accordingly, the present study develops a second-stage, activity-based workforce planning framework that complements the previously defined territorial allocation model [22]. The framework integrates predefined municipality-to-node assignments, observed municipal outpatient cardiology activity, procedure-specific scheduling times, and alternative assumptions regarding annual clinical availability, consistent with workload- and utilization-based approaches to health workforce planning [2,3,17,18,19]. Its methodological novelty lies not in proposing a new general optimization model, but in adding a transparent and auditable workforce-translation step that converts these inputs into node-specific annual and weekly workload estimates and nominal 36 h weekly specialist-schedule equivalents. The aim is to estimate the specialist working time associated with the observed outpatient activity assigned to each hub or spoke. The resulting outputs represent theoretical, activity-derived workload requirements under the model assumptions and should not be interpreted as direct measures of underlying population demand, epidemiological need, unmet need, or operationally validated staffing standards.

## 2. Materials and Methods

### 2.1. Study Setting, Data Sources, and Reference Territorial Configuration

This study was designed as a second-stage workforce planning analysis based on a previously defined territorial allocation framework.

Using outpatient cardiology as an empirical case study, we estimated specialist working-hour requirements within the hub-and-spoke territorial network of local health authority (ASL 1) Avezzano-Sulmona-L’Aquila in the Province of L’Aquila, Abruzzo, Italy. The first-stage territorial framework assigned municipalities to pre-identified hub-and-spoke centers using the 2021 ISTAT municipality-level origin–destination road travel-time matrix and population-based planning constraints. In the present study, this allocation served as the reference catchment structure for aggregating observed outpatient cardiology activity and estimating center-level workload. The reference network included three hub centers, Avezzano, L’Aquila, and Sulmona, and eight spoke centers, Carsoli, Castel di Sangro, Castelvecchio Subequo, Civitella Roveto, Montereale, Rocca di Mezzo, San Demetrio ne’ Vestini, and Trasacco. The baseline 30 min territorial configuration adopted in the previously published allocation study served as the reference scenario for workload aggregation and specialist working-hour estimation [22]. The geographic location of the study area and the spatial distribution of hub-and-spoke centers within the Province of L’Aquila are shown in Supplementary Figure S1.

The present second-stage analysis integrated three main inputs: the predefined municipality-to-node assignment dataset derived from the first-stage territorial model, anonymized ASL 1 outpatient cardiology administrative data, and standardized scheduling durations for each outpatient cardiology procedure. The outpatient cardiology administrative data were provided internally by ASL 1 Avezzano-Sulmona-L’Aquila under institutional authorization documented in note no. 0060186/25, dated 31 March 2025. Before access by the research team, the data had been anonymized and aggregated at the municipal level and contained procedure counts by municipality of residence and service type, without individual-level identifiers. The underlying territorial assignments had been generated using the 2021 ISTAT municipality-level origin–destination road travel-time matrix and population data [22]. Outpatient activity data were available at the municipal level and included procedure counts by municipality of residence and service type. Because the analysis used anonymized municipality-level data and generated only aggregate center-level outputs, no individual-level identifiable information was used. Standardized scheduling durations were obtained from the ASL 1 outpatient scheduling framework for cardiology services. These durations represented the administrative scheduling standards in use for the study rather than periodically updated empirical measurements of actual consultation or procedure times. The corresponding values are reported in Supplementary Table S1. For workforce estimation, 2019 was used as the baseline year. This choice was made because 2019 represented the last full pre-pandemic year of outpatient activity and therefore provided a stable baseline not directly affected by COVID-19-related service disruptions, temporary reductions in elective outpatient care, or subsequent recovery dynamics. The present analysis should therefore be interpreted as a proof-of-concept, activity-based planning model based on pre-pandemic observed service volumes. Its outputs represent the workload implied by the 2019 activity profile and should not be interpreted as estimates of current outpatient cardiology utilization, underlying population demand, epidemiological need, unmet need, or current waiting-list pressure.

### 2.2. Phase 1: Territorial Linkage and Catchment-Level Activity Attribution

Municipal-level outpatient cardiology activity was merged with the predefined municipality-to-node assignment dataset derived from the first-stage territorial model. No new territorial allocation was performed in the present study. All recorded outpatient cardiology procedure volumes for residents of each municipality were attributed to the corresponding assigned hub or spoke and subsequently aggregated at the center level.

### 2.3. Phase 2: Procedure-Based Workload Estimation

**Figure 1:**
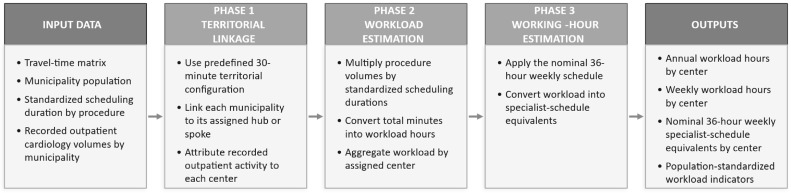
An analytical workflow for estimating outpatient cardiology workload and translating it into specialist working-hour estimates within the baseline 30 min hub-and-spoke network. Recorded municipal outpatient activity was attributed to each center, converted into workload using standardized scheduling durations, and translated into nominal 36 h weekly specialist-schedule equivalents.

For each municipality and procedure type, workload was calculated by multiplying the observed procedure volume by the corresponding standardized scheduling duration. Workload minutes were summed across all outpatient cardiology procedures and across the municipalities assigned to each hub or spoke, generating the total center-level workload. Data preparation, linkage, aggregation, and workload calculations were performed in Python 3.13.13 using pandas 2.3.3 and openpyxl 3.1.5 [26,27,28]. The complete analytical workflow is summarized in Figure 1, while the analytical phases, calculations, and corresponding outputs are detailed in Supplementary Table S4.

### 2.4. Phase 3: Translation of Workload into Specialist Working-Hour Estimates

Center-level workload was converted from minutes into annual specialist hours by dividing the total workload minutes by 60. Annual workload hours were then divided by 52 to estimate average weekly workload hours. Weekly workload was divided by the nominal 36 h weekly specialist schedule, equivalent to 1872 annual hours, to calculate 36 h weekly specialist-schedule equivalents. These values represent theoretical, activity-derived clinical-time equivalents under the model assumptions and should not be interpreted as direct staffing prescriptions or operationally validated staffing standards.

To compare the relative intensity of observed outpatient cardiology activity across centers, three population-standardized indicators were also calculated: procedures per 1000 catchment inhabitants, annual workload hours per 1000 catchment inhabitants, and nominal 36 h weekly specialist-schedule equivalents per 10,000 inhabitants. Catchment population was defined as the resident population of all municipalities assigned to each hub or spoke in the reference territorial configuration.

Overall, the study adopted a pragmatic, activity-based workforce planning approach based on observed outpatient service use and standardized scheduling times. The resulting estimates should not be interpreted as direct measures of underlying population demand, epidemiological need, unmet need, current waiting-list pressure, or operationally validated staffing standards [2,3,17,18,29].

### 2.5. Validation Scope and Sensitivity Analyses

The reference hub-and-spoke nodes and their catchments represent a model-based territorial planning configuration rather than an already implemented outpatient cardiology network. Accordingly, the present analysis was designed to estimate the specialist clinical time required if observed municipal activity was organized according to these modeled catchments. Because the proposed network had not been implemented in this form, no directly comparable node-specific benchmark corresponding to the modeled configuration was available for actual cardiologist staffing, contracted or delivered outpatient hours, waiting-list performance, or other service-capacity indicators. The resulting estimates should therefore be interpreted as theoretical, activity-derived workload requirements rather than as operationally validated staffing standards. The two sensitivity analyses assess the effects of alternative assumptions and should not be interpreted as providing external validation of the model.

Because nominal contractual hours do not necessarily align with effective outpatient clinical availability, an exploratory sensitivity analysis was conducted using alternative assumptions about annual clinical availability. In addition to the nominal 36 h weekly schedule (1872 annual hours), three scenario-based assumptions were considered: 1454, 1300, and 1200 effective annual outpatient clinical hours per cardiologist. The 1454 h scenario corresponds to the national reference value for outpatient ambulatory activity established by the Italian Ministry of Health [30] for medical specialists. The lower thresholds were retained as more conservative sensitivity assumptions to account for annual leave, training, sickness absence, administrative duties, and other non-outpatient clinical activities. Scenario-adjusted cardiologist equivalents were calculated by dividing the estimated annual workload by the assumed annual clinical availability for each scenario.

To assess uncertainty associated with using standardized rather than empirically observed procedure durations, an additional deterministic one-way sensitivity analysis was conducted. All standardized procedure times were varied simultaneously by −20%, −10%, +10%, and +20% relative to the baseline values. For each scenario, annual and weekly workload hours, nominal 36 h weekly specialist-schedule equivalents, and cardiologist equivalents under the 1454 h annual clinical-availability assumption were recalculated. These scenarios were intended to quantify the model’s proportional sensitivity to procedure-time assumptions and should not be interpreted as empirically derived uncertainty intervals.

## 3. Results

Under the baseline 30 min territorial configuration, outpatient cardiology activity recorded for residents of the study area was fully aggregated across the hub-and-spoke network. As shown in Table 1, the reference network covered a catchment population of 286,832 residents and accounted for 44,039 outpatient cardiology procedures, corresponding to an estimated 763,185 min or 12,719.8 annual workload hours. In absolute terms, activity and workload were unevenly distributed across centers. The highest values were observed in the hubs of L’Aquila and Sulmona, which accounted for 10,476 procedures and 3014.9 annual workload hours, and 10,161 procedures and 2894.3 annual workload hours, respectively. Among the spoke centers, Trasacco had the highest observed workload, with 6256 procedures and 1775.5 annual workload hours, whereas the lowest absolute workloads were observed in San Demetrio ne’ Vestini, Castelvecchio Subequo, and Civitella Roveto.

**Figure 2:**
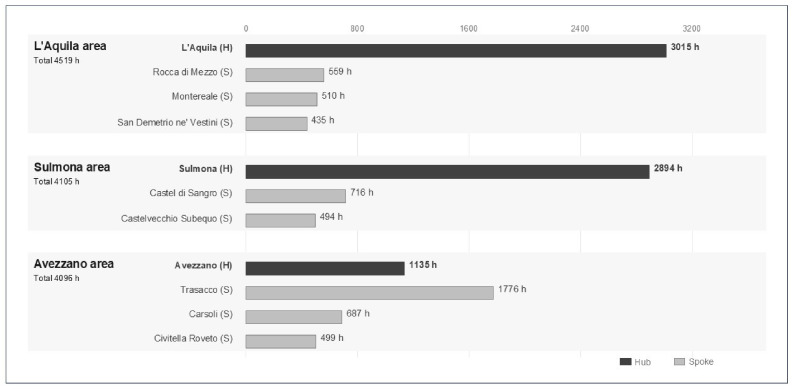
Estimated annual outpatient cardiology workload by hub-and-spoke center under the baseline 30 min territorial configuration. Centers are grouped by hub area, with spoke centers ordered by decreasing annual workload. Values are rounded to the nearest whole hour for readability; exact values are reported in Table 1.

When the annual workload was translated into weekly workload and specialist-schedule equivalents, the network generated an estimated 244.6 weekly workload hours, equivalent to 6.79 nominal 36 h weekly specialist-schedule equivalents under the model assumptions. As reported in Table 2, the largest requirements were observed in the L’Aquila and Sulmona hubs, with 58.0 and 55.7 weekly workload hours, corresponding to 1.61 and 1.55 nominal 36 h weekly specialist-schedule equivalents, respectively. The Trasacco spoke showed the greatest requirement among spoke centers, with 34.1 weekly workload hours, corresponding to 0.95 nominal 36 h weekly specialist-schedule equivalents. Overall, most of the estimated specialist working-hour requirements under the activity-based scenario were concentrated in the two main hubs and, among spoke centers, in Trasacco, indicating that workload-based specialist requirements were highly uneven across the territorial network. This distribution is also illustrated in Figure 2.

Population-standardized indicators revealed substantial variation in workload intensity across catchments. As shown in Table 3, across the network, average activity levels were 153.5 procedures per 1000 inhabitants, 44.3 workload hours per 1000 inhabitants, and 0.24 nominal 36 h weekly specialist-schedule equivalents per 10,000 inhabitants. The highest standardized values were observed in the Sulmona hub, with 251.9 procedures per 1000 inhabitants, 71.8 workload hours per 1000 inhabitants, and 0.38 specialist-schedule equivalents per 10,000 inhabitants. Among spoke centers, Castelvecchio Subequo and Trasacco showed the highest relative intensity of activity, with 246.1 and 197.3 procedures per 1000 inhabitants and 69.6 and 56.0 workload hours per 1000 inhabitants, respectively. By contrast, the Avezzano hub showed the lowest standardized activity, with 79.0 procedures per 1000 inhabitants, 23.5 workload hours per 1000 inhabitants, and 0.13 specialist-schedule equivalents per 10,000 inhabitants. These standardized differences should be interpreted as differences in observed workload intensity across catchment areas, rather than as direct measures of underlying population demand, epidemiological need, or unmet need.

In the exploratory annual clinical-availability sensitivity analysis, the total outpatient cardiology workload of 12,719.8 annual hours corresponded to 6.79 nominal specialist-schedule equivalents when the full 36 h weekly schedule was assumed to be available for outpatient activity, equivalent to 1872 annual hours. Under the national reference assumption of 1454 effective annual outpatient clinical hours, the estimated requirement increased to 8.75 cardiologist equivalents, approximately 28.7% above the nominal-schedule estimate. The corresponding estimates were 9.78 equivalents under the 1300 h scenario and 10.60 equivalents under the 1200 h scenario, approximately 44.0% and 56.0% above the nominal estimate, respectively (Supplementary Table S2). These scenarios show that the conversion of a fixed workload into workforce equivalents is materially influenced by the amount of annual working time assumed to be effectively available for outpatient activity.

The additional procedure-duration sensitivity analysis showed a proportional effect on the estimated workforce requirements. A 10% reduction in standardized procedure times decreased the annual workload from 12,719.8 to 11,447.8 h and the nominal 36 h weekly specialist-schedule equivalents from 6.79 to 6.12, whereas a 10% increase raised these estimates to 13,991.7 h and 7.47 equivalents. Under the wider ±20% scenarios, the annual workload ranged from 10,175.8 to 15,263.7 h, corresponding to 5.44–8.15 nominal specialist-schedule equivalents. Under the 1454 h annual clinical-availability assumption, the corresponding estimates ranged from 7.00 to 10.50 cardiologist equivalents (Supplementary Table S3).

Taken together, these findings indicate that estimated specialist working-hour requirements under the activity-based scenario varied not only with the size of the population served but also with the relative intensity of observed outpatient cardiology activity within each catchment area. A population-based planning criterion alone would therefore not have fully captured the heterogeneity in observed workload across the territorial network. As summarized in Table 1, Table 2 and Table 3, a limited number of nodes absorbed most of the outpatient cardiology workload, while several spoke centers were associated with small but non-negligible working-hour requirements. From a planning perspective, this means that specialist working-hour requirements can be estimated more transparently by combining observed outpatient activity with procedure-specific workload, rather than inferring them from catchment population alone.

## 4. Discussion

This study demonstrates that an activity-based workforce planning model can translate a hub-and-spoke territorial configuration into estimated specialist working-hour requirements for outpatient cardiology. Building on the previous territorial allocation framework developed for ASL 1 Avezzano-Sulmona-L’Aquila, the analysis adds an operational workforce layer to the network’s spatial design [22]. The contribution is twofold: first, it preserves the hub-and-spoke model’s territorial logic; second, it estimates the specialist clinical time associated with each node under a defined activity-based scenario. This step is relevant for implementing community health centers because territorial accessibility and infrastructure do not automatically translate into service capacity unless professional time is explicitly planned [4,10,14].

The results indicate that outpatient cardiology workload was unevenly distributed across the network. In absolute terms, most of the estimated workload was concentrated in the L’Aquila and Sulmona hubs, followed by the Trasacco spoke. This pattern aligns with the expected role of hubs as major service nodes within hub-and-spoke service delivery models [22,31]. However, it also shows that selected spoke centers may absorb substantial volumes of specialist outpatient activity. In a geographically complex territory, this is an important planning finding: spoke centers should not be viewed solely as peripheral access points but also as operational nodes that may require dedicated specialist time to maintain proximity and continuity of care.

Population-standardized indicators provided a more nuanced picture. Sulmona and Castelvecchio Subequo showed the highest standardized workload values, while Avezzano showed the lowest. These differences indicate that workload intensity was not simply proportional to catchment population size. The higher standardized workload observed in Sulmona and Castelvecchio Subequo may reflect local patterns of service use, procedure mix, demographic structure, or historical referral pathways. Within this model, these values should be interpreted as workload signals: they identify where specialist time would be required if the network was organized around the observed pre-pandemic service-use profile.

These findings reinforce the limits of planning outpatient specialist services using population-based criteria alone. Population catchments are essential for defining territorial responsibility and potential coverage, but they do not capture the volume, type, or time-intensity of services delivered. Two catchments with similar population sizes may generate different specialist workloads because of differences in procedure mix, referral behavior, patient flows, historical service organization, or local accessibility. This aligns with the health workforce planning literature, which shows that headcounts and provider-to-population ratios are insufficient when used in isolation and should be complemented by workload, service utilization, geography, models of care, and local organizational arrangements [2,3,17,18,19].

The proposed model also contributes to the implementation debate around DM 77/2022. The reform introduced a territorial care architecture based on community health centers, community hospitals, and territorial operations centers, but translating these structures into operational capacity remains a key challenge [4,7,10,12]. This issue is particularly relevant for outpatient specialist care. Recent Italian evidence has emphasized that outpatient specialists are a core component of community healthcare, but that available data on specialist workforce input, delivered hours, booked visits, waiting-list dynamics, and effective service capacity remain limited [15]. By converting observed outpatient activity into estimated specialist working hours, the present model provides a practical tool for addressing part of this operational gap.

The territorial context of ASL 1 further supports the relevance of linking spatial planning with workforce planning. Rural, inland, and mountainous areas face access barriers that are not limited to distance from facilities; they also include travel time, transport conditions, settlement dispersion, and workforce availability [3,5,6,21]. In such settings, assigning municipalities to hubs and spokes is only the first planning step. A second step is needed to estimate the specialist working time associated with each node. The proposed approach connects these two dimensions by combining municipal catchments with procedure-specific workload.

Cardiology represents an appropriate first application of this second-stage planning approach. Cardiovascular diseases remain a major cause of morbidity, mortality, hospital admissions, and healthcare expenditure in Europe, and their management requires continuity across prevention, primary care, specialist assessment, diagnostics, follow-up, and post-acute management [23,24]. In community settings, outpatient cardiology supports chronic disease monitoring, risk stratification, timely referral, and continuity after acute events. Italian evidence has also shown territorial variation in outpatient cardiovascular visit rates and waiting times, suggesting that local service capacity and organizational choices may influence equitable access to cardiology services [20]. The organization of cardiology outpatient services and heart failure clinics in Italy further highlights the importance of structured links between specialist care, general practice, caregivers, and local services. The present study complements this literature by focusing on the specialist working time implied by outpatient cardiology activity within a predefined territorial network [32].

The annual clinical-availability sensitivity analysis has direct operational implications. The nominal scenario assumes that the entire contractual schedule can be devoted to outpatient activity and therefore produces the lowest workforce estimate. By contrast, the national reference assumption of 1454 annual outpatient clinical hours may provide a more appropriate policy-reference scenario when part of physicians’ working time is allocated to leave, training, administrative duties, care coordination, or other clinical functions. The 1300 and 1200 h scenarios represent progressively more conservative planning assumptions. These scenarios should not be interpreted as alternative forecasts or validated staffing standards; rather, they show how local organizational assumptions regarding effective clinical availability affect the conversion of workload hours into workforce equivalents. Health authorities should therefore select and explicitly document the annual clinical-availability assumption that most closely reflects local contractual arrangements and models of care.

Overall, the study suggests that hub-and-spoke planning for community health centers should include a workforce translation step. A first-stage allocation model can define which municipalities are assigned to each hub or spoke under travel-time and population constraints. A second-stage activity-based workload model can then estimate how much specialist time is associated with each node. This two-step approach is particularly useful in low-density and mountainous areas, where the feasibility of territorial care depends on both proximity and workforce availability [3,22]. The calculation framework is transparent and auditable, but its transferability depends on preserving its analytical sequence rather than reproducing the specific territorial configuration or numerical parameters used in the present case study. In another Italian region, the model could be reapplied by defining locally relevant service nodes and catchment areas, mapping municipality-level or equivalent outpatient activity to those catchments, linking each procedure to locally approved or empirically observed delivery times, and converting the resulting workload into specialist working-hour equivalents using applicable regional organizational and contractual assumptions. Within the common framework established by DM 77/2022, regional implementation would therefore require local calibration of the territorial network, service portfolio, referral pathways, public–private service distribution, and effective annual clinical availability.

The same analytical logic could be adapted to international healthcare systems, although community health center and hub-and-spoke categories would need to be replaced with corresponding local organizational units. Procedure coding systems, referral rules, workforce contracts, professional skill mix, patient-flow patterns, and annual clinical-time assumptions would also need to be recalibrated. Thus, the transferable component is the modular workload-estimation framework, whereas the catchment configuration, activity data, procedure-time coefficients, and workforce assumptions remain context-specific inputs. Full reproduction of the present case-study outputs is conditional on authorized access to the underlying outpatient activity data, municipality-to-node assignment files, and the analytical materials described in the Data Availability Statement.

The findings have practical implications for territorial health planning and the implementation of DM 77/2022. Local health authorities could apply the framework as part of a stepwise planning process. First, the relevant service nodes, catchment areas, and outpatient service portfolio would be defined. Second, updated activity volumes and procedure-time assumptions would be used to estimate annual and weekly specialist working-hour requirements for each hub or spoke. Third, these estimates could be compared with existing staffing, contracted or delivered outpatient hours, and available service capacity to identify potential shortages, surpluses, or opportunities for redistribution across the network.

For budgeting purposes, the estimated working-hour requirements could be converted into locally applicable expenditure scenarios using the relevant employment contracts, salary costs, sessional tariffs, or service-purchasing arrangements. The model could therefore support comparisons between alternative organizational options, including redistribution of existing professional time, recruitment of additional staff, rotational coverage across nodes, or phased activation of specialist services. However, the framework does not itself determine the preferred organizational or financial solution, which would also require consideration of clinical priorities, equity, workforce availability, and local budget constraints.

Within the future implementation of DM 77/2022, the model could be used to prioritize the activation or strengthening of selected nodes, assess the workload consequences of alternative territorial configurations, and update workforce estimates as new activity, staffing, waiting-list, and service-performance data become available. In this way, activity-based workload estimation can complement accessibility-based planning and provide health authorities with a transparent operational input for workforce allocation and budget planning in geographically complex areas [13,14,22].

The second-stage workload model is computationally lightweight because it relies primarily on deterministic data linkage, aggregation, and arithmetic conversion. Its implementation requires structured activity data, consistent territorial identifiers, an agreed procedure-time catalog, routine data-quality checks, and the ability to update the model inputs and assumptions by the personnel.

Artificial intelligence could support future extensions of the framework by analyzing larger longitudinal datasets, identifying nonlinear patterns in observed outpatient service use, updating procedure-time estimates, detecting anomalous activity patterns, and generating scenario-based workload forecasts. No AI-based methods were used in the present study, which relies on a transparent deterministic approach. Future AI applications would require external validation, explainability, appropriate data-governance safeguards, and careful distinction between observed activity, epidemiological need, and operationally validated staffing standards.

Several limitations should be acknowledged. Consistent with its activity-based design, the model estimates the specialist working time implied by observed service-use volumes rather than directly estimating underlying population demand, epidemiological need, or unmet need. Observed utilization may itself be shaped by historical service availability, referral patterns, territorial access barriers, private-sector substitution, and administrative or organizational practices; these determinants were not directly measured in the present analysis. Because 2019 was selected as the last full pre-pandemic baseline year, the estimates do not capture possible post-pandemic changes in outpatient volumes, referral pathways, waiting-list dynamics, patient behavior, or service organization, which may be relevant given documented changes in outpatient specialist care before and after the COVID-19 pandemic [15].

Accordingly, the results should be interpreted as a historical pre-pandemic activity baseline for demonstrating the workload-estimation framework, rather than as estimates of current outpatient demand, service capacity, or staffing requirements. Applying the model to present-day planning would require recalculation using updated activity volumes and contemporaneous information on service organization, workforce availability, referral patterns, and waiting-list dynamics.

Workload was calculated using standardized scheduling times rather than empirically measured consultation and procedure durations. Standardized scheduling slots may differ systematically from actual service-delivery times and may therefore introduce bias into the workload estimates. The model may underestimate specialist working-hour requirements when consultations require additional time because of clinical complexity, documentation, interruptions, or care coordination, and may overestimate them when procedures are delivered more rapidly than scheduled. The procedure-duration sensitivity analysis quantified the effect of uniform deviations from the standardized times; however, actual deviations may differ across procedure types, patients, and clinical settings. Future applications should therefore recalibrate the model using directly observed service-delivery times where available.

Other sources of uncertainty were not explicitly incorporated into the model. Clinical complexity may vary within the same procedure category, while referral pathways, seasonal fluctuations, cancelations, and patient preferences regarding provider, location, or public versus private care may influence both the volume and territorial distribution of observed activity. These factors are not captured by the deterministic sensitivity analyses and should be considered in future applications using more detailed case-mix, referral, patient-flow, and time-specific activity data.

Finally, this proof-of-concept analysis was limited to one local health authority and one specialty, and the modeled hub-and-spoke configuration had not yet been implemented as an outpatient cardiology network. Direct operational validation against node-specific staffing, contracted or delivered outpatient hours, waiting-list indicators, or other service-performance measures was therefore not possible because no fully corresponding real-world organizational benchmark was available. Future validation could be conducted prospectively following pilot implementation of the proposed network. Alternatively, existing facilities, cardiologist staffing, delivered clinical hours, patient flows, and waiting-list indicators could be mapped to the proposed catchments, allowing observed service capacity to be compared with the model-based workload requirements.

## 5. Conclusions

This study demonstrates that an activity-based workforce planning model can translate a hub-and-spoke territorial configuration into estimated outpatient specialist working-hour requirements. Building on a previously defined allocation framework for ASL 1 Avezzano-Sulmona-L’Aquila, the model adds an operational workforce layer by linking municipal outpatient cardiology activity, standardized scheduling durations, catchment assignments, and specialist scheduling assumptions.

The findings show that outpatient cardiology workload was unevenly distributed across the network and that workload intensity was not proportional to catchment population size. This indicates that population-based and travel-time planning criteria should be complemented by workload-based estimates when planning outpatient specialist services in community settings.

By converting observed service-use volumes into estimated specialist working hours and 36 h weekly specialist-schedule equivalents, the model provides a transparent, activity-derived workload framework to support the operational planning of community health centers. These estimates reflect the recorded 2019 service-use profile and should not be interpreted as direct measures of current population demand, epidemiological need, or unmet need. In low-density and mountainous areas, where access depends on both proximity and workforce availability, this approach can help bridge the gap between territorial allocation and the professional time required to make hub-and-spoke networks functional.

The resulting working-hour estimates may also provide an operational basis for comparing existing capacity with modeled requirements, developing locally calibrated staffing and budget scenarios, and prioritizing the phased activation of specialist services within the DM 77/2022 framework.

Although applied here to outpatient cardiology in a single local health authority, the modular framework may be adapted to other specialties, territories, and healthcare systems. Such applications would require local definition of service nodes and catchments and recalibration of procedure-time coefficients, referral pathways, workforce policies, and effective clinical-time assumptions.

Future applications should use updated activity data and undertake prospective or structured operational validation by comparing model-based estimates with actual staffing, delivered outpatient hours, waiting-list indicators, and other service-performance measures mapped to the proposed catchments.

## Figures and Tables

**Table 1:** Aggregated outpatient cardiology activity and estimated annual workload by hub-and-spoke center.

Center	Type	Catchment Population	Procedures (n)	Estimated Workload (Hours)	Share of Total Workload (%)
Avezzano	Hub	48,319	3815	1134.8	8.9
Carsoli	Spoke	15,234	2346	687.1	5.4
Civitella Roveto	Spoke	17,814	1679	498.7	3.9
Trasacco	Spoke	31,706	6256	1775.5	14.0
L’Aquila	Hub	69,717	10,476	3014.9	23.7
Montereale	Spoke	13,101	1800	510.1	4.0
Rocca di Mezzo	Spoke	16,797	1989	559.3	4.4
San Demetrio ne’ Vestini	Spoke	12,362	1567	434.7	3.4
Sulmona	Hub	40,334	10,161	2894.3	22.8
Castel di Sangro	Spoke	14,349	2203	715.9	5.6
Castelvecchio Subequo	Spoke	7099	1747	494.4	3.9
Overall Network	-	286,832	44,039	12,719.8	100.0

Note. Outpatient cardiology procedures were aggregated by assigned hub or spoke center under the baseline 30 min territorial configuration. Workload was estimated by summing standardized procedure times across all outpatient cardiology services attributable to each center. Totals may differ slightly because of rounding.

**Table 2:** Estimated outpatient cardiology working-hour requirements by hub-and-spoke center.

Center	Type	Estimated Annual Workload (Hours)	Estimated Weekly Workload (Hours)	36 h Weekly Specialist-Schedule Equivalents
Avezzano	Hub	1134.8	21.8	0.61
Carsoli	Spoke	687.1	13.2	0.37
Civitella Roveto	Spoke	498.7	9.6	0.27
Trasacco	Spoke	1775.5	34.1	0.95
L’Aquila	Hub	3014.9	58.0	1.61
Montereale	Spoke	510.1	9.8	0.27
Rocca di Mezzo	Spoke	559.3	10.8	0.30
San Demetrio ne’ Vestini	Spoke	434.7	8.4	0.23
Sulmona	Hub	2894.3	55.7	1.55
Castel di Sangro	Spoke	715.9	13.8	0.38
Castelvecchio Subequo	Spoke	494.4	9.5	0.26
Overall Network	-	12,719.8	244.6	6.79

Note. Weekly specialist-schedule equivalents were calculated by dividing estimated weekly workload hours by 36, according to the specialist scheduling model adopted in the original planning framework. These values represent theoretical clinical-time equivalents under the model assumptions and should not be interpreted as direct staffing prescriptions.

**Table 3:** Standardized outpatient cardiology activity and workload indicators by hub-and-spoke center.

Center	Type	Catchment Population	Procedures (per 1000 Inhabitants)	Workload (hours per 1000 Inhabitants)	36 h Weekly Specialist-Schedule Equivalents per 10,000 Inhabitants
Avezzano	Hub	48,319	79.0	23.5	0.13
Carsoli	Spoke	15,234	154.0	45.1	0.24
Civitella Roveto	Spoke	17,814	94.3	28.0	0.15
Trasacco	Spoke	31,706	197.3	56.0	0.30
L’Aquila	Hub	69,717	150.3	43.2	0.23
Montereale	Spoke	13,101	137.4	38.9	0.21
Rocca di Mezzo	Spoke	16,797	118.4	33.3	0.18
San Demetrio ne’ Vestini	Spoke	12,362	126.8	35.2	0.19
Sulmona	Hub	40,334	251.9	71.8	0.38
Castel di Sangro	Spoke	14,349	153.5	49.9	0.27
Castelvecchio Subequo	Spoke	7099	246.1	69.6	0.37
Overall Network	-	286,832	153.5	44.3	0.24

Note. Standardized indicators were calculated using the catchment population assigned to each center under the baseline 30 min territorial configuration.

## Data Availability

Municipal demographic data were obtained from the Italian National Institute of Statistics (ISTAT). The 2021 national origin–destination road travel-time matrix is publicly available from the ISTAT “Matrices of Contiguity, Distance, and Commuting” repository (https://www.istat.it/notizia/matrici-di-contiguita-distanza-e-pendolarismo/, accessed on 2 August 2026). The municipal-level outpatient cardiology activity data were internally provided by ASL 1 Avezzano-Sulmona-L’Aquila under institutional authorization documented in note no. 0060186/25, dated 31 March 2025, and are not publicly available. Standardized procedure-time information is reported in Supplementary Table S1. The outpatient activity dataset, municipality-to-node assignment files, processed analytical datasets, analytical scripts used for data processing, workload calculation, and sensitivity analyses, and other algorithm-related materials may be made available by the corresponding authors upon reasonable request, subject to prior authorization by ASL 1 Avezzano-Sulmona-L’Aquila and compliance with the applicable institutional data-sharing requirements.

## Supplementary Materials

The following supporting information can be downloaded at: https://www.mdpi.com/article/10.3390/healthcare14152435/s1, Table S1. Standardized scheduling duration for outpatient cardiology procedures used for workload estimation. Table S2. Exploratory sensitivity analysis of cardiologist-equivalent requirements under alternative annual clinical-availability assumptions. Table S3. Sensitivity of estimated outpatient cardiology workload and cardiologist equivalents to alternative standardized procedure-duration assumptions. Table S4. Analytical phases, calculations, and outputs of the activity-based workforce model. Figure S1. Geographic location of the study area and hub-and-spoke Community Health Center network in the Province of L’Aquila (ASL 1 Avezzano-Sulmona-L’Aquila), Abruzzo, Italy. The left panel shows the location of the Abruzzo region within Italy, with the Province of L’Aquila highlighted as the ASL 1 study area. The right panel shows the hub-and-spoke network within the province of L’Aquila under the baseline 30-min territorial configuration, comprising three hub centers (Avezzano, L’Aquila, Sulmona; filled circles) and eight spoke centers (Carsoli, Castel di Sangro, Castelvecchio Subequo, Civitella Roveto, Montereale, Rocca di Mezzo, San Demetrio ne’ Vestini, Trasacco; open circles), connected by lines indicating hub-spoke assignments. Grey shades represent the three healthcare districts: L’Aquila area, Marsica area and Peligno-Sangrina area.

## Abbreviations

ASL: Local health authority (Azienda Sanitaria Locale); DM: Ministerial Decree; ISTAT: Italian National Institute of Statistics.
