# Supplementary material for: Activity-Based Algorithm for Translating Observed Outpatient Activity into Specialist Working-Hour Requirements Within Community Health Center Catchment Areas
Source: Healthcare (Basel). 2026 Aug 6;14(15):2435. doi: 10.3390/healthcare14152435 (PMC13465226; doi:10.3390/healthcare14152435)
Supplement: Supplementary file 1 [file healthcare-14-02435-s001.zip › healthcare-4392756-supplementary.pdf]

Supplementary Materials

Activity-Based Algorithm for Translating Observed Outpatient Activity into Specialist Working-Hour Requirements Within Community Health Center Catchment Areas

Supplementary Table S1. Standardized scheduling duration for outpatient cardiology procedures used for workload estimation

Outpatient cardiology procedure	Standardized scheduling duration (minutes)
Cardiac ultrasound/echocardiography	30
Electrocardiogram	10
Cardiology consultation	20
Cardiovascular exercise test with a cycle ergometer	30
Pediatric cardiology consultation	20
Follow-up cardiology consultation	15
24-hour ambulatory blood pressure monitoring, Holter BP	10
24-hour dynamic electrocardiographic monitoring, Holter ECG	10
Cardiac Doppler echocardiography	30
Cardiac color Doppler echocardiography	30
Follow-up cardiology control visit	15
Resting or pharmacological stress echocardiography	30
Supra-aortic trunk color Doppler ultrasound	30
Cardiovascular exercise test with a treadmill	60
Standard electrocardiogram	10
Resting cardiac color Doppler echocardiography	30
First cardiology consultation	20
Other cardiovascular exercise tests/thallium stress test	30
Follow-up pediatric cardiology control visit	15
Resting or post-exercise cardiac Doppler echocardiography	30

Note. Standardized delivery times were derived from the ASL 1 Avezzano-Sulmona-L'Aquila outpatient scheduling framework. These values represent standardized scheduling times used for workload estimation and workforce planning purposes and should not be interpreted as empirically measured consultation durations. Procedure labels reflect the administrative/service nomenclature used in the ASL 1 outpatient scheduling framework. Some procedures with similar clinical descriptions correspond to distinct administrative codes within the ASL 1 scheduling framework and are therefore listed separately.

Supplementary Table S2. Exploratory sensitivity analysis of cardiologist-equivalent requirements under alternative annual clinical-availability assumptions

Effective annual outpatient clinical availability (hours)	Cardiologist-equivalent estimate	Absolute difference from nominal scenario	Increase from nominal scenario (%)
1872 h	6.79	Reference	Reference
1454 h	8.75	+1.95	+28.5%
1300 h	9.78	+2.99	+44.0%
1200 h	10.60	+3.81	+56.0%

Note. The nominal scenario assumes that the full 36-hour weekly contractual schedule is available for outpatient activity. The 1,454-hour scenario represents the national reference value for annual outpatient clinical activity, whereas the 1,300- and 1,200-hour scenarios represent progressively more conservative assumptions. Differences and percentage increases are calculated relative to the nominal 1,872-hour scenario. These estimates are scenario-based workload equivalents and should not be interpreted as validated staffing prescriptions.

.

Supplementary Table S3. Sensitivity of estimated outpatient cardiology workload and cardiologist equivalents to alternative standardized procedure-duration assumptions.

Procedure-duration scenario	Annual workload hours	Weekly workload hours	Nominal 36-hour specialist-schedule equivalents	Cardiologist equivalents under 1,454 effective annual outpatient clinical hours
−20%	10,175.8	195.7	5.44	7.00
−10%	11,447.8	220.1	6.12	7.87
Baseline	12,719.8	244.6	6.79	8.75
+10%	13,991.7	269.1	7.47	9.62
+20%	15,263.7	293.5	8.15	10.50

Note. The baseline scenario uses the standardized procedure durations reported in Table S1 and corresponds to an aggregate workload of 763,185 minutes. In the alternative scenarios, all procedure durations were varied simultaneously by −20%, −10%, +10%, or +20%, without intermediate rounding. Weekly workload was calculated by dividing annual workload hours by 52. Nominal 36-hour weekly specialist-schedule equivalents were calculated by dividing annual workload hours by 1,872, corresponding to 52 weeks × 36 hours. Cardiologist equivalents were calculated using the national reference assumption of 1,454 effective annual outpatient clinical hours per cardiologist. These deterministic scenarios quantify the proportional sensitivity of the estimates to uniform changes in procedure-duration assumptions and should not be interpreted as empirically derived uncertainty intervals.

Supplementary Table S4. Analytical phases, calculations, and outputs of the activity-based workforce model

Analytical phase	Input	Calculation	Output
Territorial linkage	Predefined municipality-to-node assignments; municipal outpatient activity	Recorded outpatient activity for residents of each municipality was linked to the corresponding assigned hub or spoke.	Recorded activity attributed to each territorial node
Procedure workload	Procedure volume; standardized scheduling duration in minutes	Procedure volume × standardized scheduling duration	Workload minutes by municipality and procedure
Municipal workload	Workload minutes by procedure	Sum across all cardiology procedures	Total municipal workload minutes
Center-level aggregation	Municipal procedure volumes and workload; territorial assignments	Sum across municipalities assigned to each hub or spoke	Center-level procedure volume and workload minutes
Annual workload	Center-level workload minutes	Workload minutes ÷ 60	Annual workload hours
Weekly workload	Annual workload hours	Annual workload hours ÷ 52	Average weekly workload hours
Nominal specialist-schedule equivalents	Annual or weekly workload hours	Annual hours ÷ 1,872, equivalent to weekly hours ÷ 36	Nominal 36-hour weekly specialist-schedule equivalents
Procedures per 1,000 inhabitants	Center procedure volume; catchment population	Procedures ÷ population × 1,000	Population-standardized procedure volume
Workload per 1,000 inhabitants	Annual workload hours; catchment population	Workload hours ÷ population × 1,000	Population-standardized workload intensity
Nominal equivalents per 10,000 inhabitants	Nominal equivalents; catchment population	Nominal equivalents ÷ population × 10,000	Population-standardized schedule equivalents
Annual clinical-availability sensitivity	Total annual network workload; annual availability assumptions	Total annual network workload ÷ assumed annual clinical availability (1,872, 1,454, 1,300, or 1,200 hours)	Scenario-specific cardiologist equivalents
Procedure-duration sensitivity	Baseline standardized durations	All standardized scheduling durations × 0.80, 0.90, 1.00, 1.10, or 1.20; downstream calculations repeated	Scenario-specific annual and weekly workload hours, nominal specialist-schedule equivalents, and equivalents under the 1,454-hour assumption

Note. Territorial assignments were inherited from the previously developed first-stage allocation model; no new territorial allocation was performed in the present study. Both sensitivity analyses were deterministic and should not be interpreted as empirically derived uncertainty intervals.

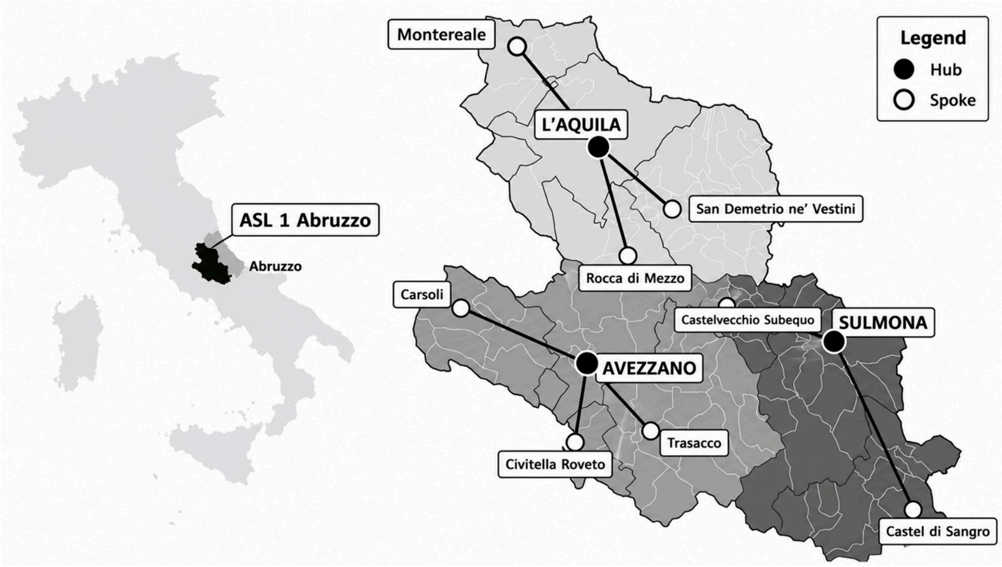

Supplementary Figure S1. Geographic location of the study area and hub-and-spoke Community Health Center network in the Province of L'Aquila (ASL 1 Avezzano-Sulmona-L'Aquila), Abruzzo, Italy. The left panel shows the location of the Abruzzo region within Italy, with the Province of L'Aquila highlighted as the ASL 1 study area. The right panel shows the hub-and-spoke network within the Province of L'Aquila under the baseline 30-minute territorial configuration, comprising three hub centers (Avezzano, L'Aquila, Sulmona; filled circles) and eight spoke centers (Carsoli, Castel di Sangro, Castelvecchio Subequo, Civitella Roveto, Montereale, Rocca di Mezzo, San Demetrio ne' Vestini, Trasacco; open circles), connected by lines indicating hub-spoke assignments. Grey shades represent the three healthcare districts: L'Aquila area, Marsica area, and Peligno-Sangrina area.
